# Post-Mastectomy Pain: An Updated Overview on Risk Factors, Predictors, and Markers

**DOI:** 10.3390/life11101026

**Published:** 2021-09-29

**Authors:** Marco Calapai, Emanuela Esposito, Luisa Puzzo, Daniele Alfio Vecchio, Rosario Blandino, Giuseppe Bova, Domenico Quattrone, Carmen Mannucci, Ilaria Ammendolia, Cristina Mondello, Sebastiano Gangemi, Gioacchino Calapai, Luigi Cardia

**Affiliations:** 1 Breast Unit, San Vincenzo Hospital, Azienda Sanitaria Provinciale Messina, 98039 Messina, Italy; calapaimarco@gmail.it (M.C.); luisa.puzzo@asp.messina.it (L.P.); daniele.vecchio@asp.messina.it (D.A.V.); rolumic@libero.it (R.B.); 2 Genetics and Pharmacogenetics Unit, Policlinico Universitario “G. Martino”, University of Messina, 98125 Messina, Italy; emanuela.esposito@unime.it; 3 Department of Chemical, Biological, Pharmacological and Environmental Sciences, University of Messina, 98125 Messina, Italy; 4 Pain Therapy Unit, San Vincenzo Hospital, Azienda Sanitaria Provinciale Messina, 98039 Messina, Italy; bovagiuseppe75@gmail.com; 5 Pain Therapy Unit, Grande Ospedale Metropolitano “Bianchi-Melacrino-Morelli”, 89133 Reggio Calabria, Italy; domenicoquattrone@gmail.com; 6 Department of Biomedical and Dental Sciences and Morphological and Functional Imaging, University of Messina, 98125 Messina, Italy; cmannucci@unime.it (C.M.); mondelloc@unime.it (C.M.); 7 Department of Clinical and Experimental Medicine, University of Messina, 98125 Messina, Italy; ilaria.ammendolia@gmail.com (I.A.); luigi.cardia@unime.it (L.C.); 8 School and Operative Unit of Allergy and Clinical Immunology, Department of Clinical and Experimental Medicine, University of Messina, 98125 Messina, Italy; sebastiano.gangemi@unime.it

**Keywords:** post-mastectomy pain, post-mastectomy pain syndrome, chronic pain, mastectomy, risk factors, predictors, biomarkers

## Abstract

After breast surgery, women frequently develop chronic post-mastectomy pain (PMP). PMP refers to the occurrence of pain in and around the area of the mastectomy lasting beyond three months after surgery. The nature of factors leading to PMP is not well known. When PMP is refractory to analgesic treatment, it negatively impacts the lives of patients, increasing emotional stress and disability. For this reason, optimizing the quality of life of patients treated for this pathology has gained more importance. On the basis of the findings and opinions above, we present an overview of risk factors and predictors to be used as potential biomarkers in the personalized management of individual PMP. For this overview, we discuss scientific articles published in peer-reviewed journals written in the English language describing risk factors, predictors, and potential biomarkers associated with chronic pain after breast surgery. Our overview confirms that the identification of women at risk for PMP is fundamental to setting up the best treatment to prevent this outcome. Clinical practice can be planned through the interpretation of genotyping data, choosing drugs, and tailoring doses for each patient with the aim to provide safer and more effective individual analgesic treatment.

## 1. Introduction

Breast cancer is commonly diagnosed in women around the world [1]. However, in recent decades, as a result of progress in breast cancer management, survival has significantly increased. Unfortunately, severe post-surgical pain is generally accompanied by a substantial risk of developing chronic pain [2]. Indeed, it has been observed that, after breast surgery, women develop severe post-surgery pain and chronic post-mastectomy pain (PMP); among surviving women, from 25% to 60% of those who have been subjected to mastectomy for breast cancer suffer from post-surgery pain [3,4]. Other epidemiological data show that about half of all women undergoing mastectomy for breast cancer experience chronic pain, with 25% developing moderate-to-severe pain [5]. Chronic post-mastectomy pain (PMP) is the main symptom of a syndrome referring to the occurrence of pain in and around the area of mastectomy lasting beyond three months after surgery when all other possible causes of pain are ruled out [6,7]. It may last for years or be present for life [8]. The nature of factors leading to PMP is not well known, though it has been thought that youth and surgery, including lymphadenectomy, could be predisposing factors, and others, such as anatomical, psychosocial, and genetic factors, could be considered as additional issues [9,10]. Chronic PMP comprises phantom breast pain, cicatrix pain, and neuropathic pain produced by injury to skin nervous tissue trapped in cicatrix tissue. Other times, PMP can be because of a capsule formed following reconstruction or by lateral and medial pectoral nerves compressed by the pectoralis muscle, and to the detachment of the muscle serratus anterior compressed by the implant [11]. This condition is characterized by continuing pain, generally reported as “burning” and/or “shooting”, associated with palpable allodynia [12]. PMP was considered in the past to be an infrequent problem, but more recently, it has been evaluated to be present in 13.5–25% of women who have undergone total or partial surgical breast excision, independent of mammary reconstruction [13]. When PMP is refractory to analgesic treatment, it negatively impacts the lives of patients, increasing emotional stress and disability, as occurs in general for chronic pain [14]. For this reason, optimizing the quality of life of patients treated for this pathology has gained more importance [15]. In general, pain persistency has a significant economic burden for healthcare systems [16]. Moreover, we have incomplete knowledge concerning the specific risk factors causing chronicization of PMP; this hinders research into the correct management of this condition and the application of adequate treatment and prevention [6]. Regarding management of pain, on the basis of its partial neuropathic origin, it has been observed that both gabapentinoids [17] and tricyclic antidepressants [18] reduce chronic pain intensity in patients undergoing surgery. Furthermore, standard therapies used for post-surgery pain, represented principally by opioids and non-steroidal anti-inflammatory drugs, cannot be sufficiently effective when prescribed after surgery to patients vulnerable to neuropathic pain, and treatments often require hours to be effective [19]. Finally, anatomical breast research shows the locations of nerves at risk during mastectomy, suggesting the development of a map for breast surgery to reduce nerve damage. Moreover, targeted muscle reinnervation and regenerative peripheral nerve interfaces were indicated for the prevention of neuroma formation after nerve transection with axillary dissection and mastectomy [20]. On the basis of the findings and opinions above, we present an overview of risk factors and predictors to be used as potential biomarkers in the personalized management of individual PMP.

## 2. Materials and Methods

For this overview, we collected scientific articles published in peer-reviewed journals written in the English language describing risk factors, predictors, and potential biomarkers associated with chronic pain after breast cancer surgery. We used the criteria of the International Association for the Study of Pain (IASP) to define persistent postsurgical pain as “pain that develops after surgical intervention and lasts at least 2 months, with exclusion of other potential causes for the pain” [7]. A search was performed independently by two researchers (blinded to the authors and, initially, on results) in the major scientific databases and search engines of peer-reviewed literature on life sciences and biomedical topics (PubMed, Scopus, Embase, Web of Science, Google Scholar) by using a combination of the keywords “pain”, “post-mastectomy”, “post mastectomy pain”, post mastectomy pain syndrome”, “breast surgery”, “markers”, “biomarkers”, “risk factors”, and “predictors” in titles and abstracts, starting from January 1996 to June 2021. All full-text articles were retrieved, and the references were manually searched. In addition, reference lists of narrative reviews and expert opinions were also searched in an attempt to locate more studies describing the potentiality of biomarkers for the management of PMP. All duplicates were removed. The reviewers resolved disagreements by discussion.

## 3. Results

### 3.1. Pain Biomarkers in Breast Cancer

Pain is a common symptom in women affected by breast cancer. Before analyzing PMP, we briefly summarize the main principal findings aimed to identify potential biomarkers associated with pain in this oncologic condition. In breast cancer patients, it has been noted that pain is often linked with other issues such as fatigue and depression. This leads us to hypothesize that the co-presence of these symptoms could be due to a shared pathogenetic mechanism based on inflammation [21,22]. In particular, it has been observed that co-morbidity of depression and chronic pain is highly prevalent [23], and it has been proposed that chronic inflammation is a common mediator of these co-morbidities [24].

This aspect was investigated by evaluating the possible correlation of these symptoms with blood concentrations of noradrenaline and adrenaline as an index for sympathetic nervous system activity and cortisol and adrenocorticotropic hormone for hypothalamic–pituitary–adrenal activity in 104 women with advanced breast cancer. Simultaneous enhancement of noradrenaline, adrenaline, adrenocorticotropic hormone, and cortisol was observed with evidence of pain, depression, and fatigue occurring in the women recruited for the study [25]. This indicates that the occurrence of pain and fatigue is related to depression, suggesting that noradrenaline and adrenaline, together with cortisol and adrenocorticotropic hormone, could be used as predictors for pain in breast cancer. The relationship between inflammation and breast cancer pain associated with fatigue, anxiety, sleep, and mood disorders was also studied in women in the early stage of the disease. In this case, it was shown that women reporting pain had higher levels of C-reactive protein and interleukin-13 and more interference of pain with quality of life, together with a higher incidence of depression and sleep disorders, in comparison with the same parameters evaluated in women without pain. The study results were interpreted as a strengthening of the notion of a link between activation of the immune system, inflammation, and occurrence of pain [26]. Recently, data from the study named TRIUMPH were collected to assess the efficacy of the bisphosphonate drug pamidronate, used for the therapy of metastatic breast cancer, on 57 women with pain grading from low to moderate. As a secondary outcome, the study aimed to investigate the relationship between serum cytokines and pain evaluated through the application of the Brief Pain Inventory (BPI) questionnaire, a self-administered questionnaire designed to assess cancer pain [27]. Following chemical analysis, a significant relationship was found between pain scores obtained via the BPI and cytokines such as IL-1β, IL-2, IL-4, IL-5, IL-12p70, IL-17A, IL-23, granulocyte-macrophage colony-stimulating factor (GM-CSF), and interferon (IFN)-γ. These findings led the authors to suggest monitoring of the above cited cytokines, using them as predictors for the severity of pain in breast cancer [28]. C-reactive protein, measured before and after treatment, was investigated through a retrospective study on 366 women affected by breast cancer for its possible role as a pain predictor for potential different responders by ethnicity to radiotherapy. The results showed that scores for pain were ethnically different, and in particular, their increase was associated with the greatest levels of this protein before radiotherapy and more pronounced in women with breast cancer and obesity [29].

### 3.2. Risk Factors, Predictors, and Pain Biomarkers in PMP

Pain management is usually conducted by paying attention to the perception of pain as patients recognize it through self-evaluation. Thus, self-evaluation by patients of pain intensity and pain interference (defined as the results obtained with answers to questions about daily activities) is frequently used as a marker guiding therapy [30]. Either the interference or the intensity is evaluated by employing scales or questionnaires such as visual analogue scale (VAS) and/or the numerical rating scale (NRS). However, the usefulness of these modalities to measure pain is limited because they are characterized by strong subjectivity [31]. Knowledge of risk factors, predictors, and potential biomarkers is mandatory to improve the management of pain.

#### 3.2.1. Risk Factors

Preoperative characteristics more frequently related to PMP were investigated through psychosocial and psychophysical assessment in 259 women undergoing breast surgery. The results showed that pre-existing surgical area pain, less schooling, frequent somatization, sleep disorders, and axillary dissection are variables useful as predictors of more severe pain [32] (Table 1). Psychological factors have also been associated with persistent pain after mastectomy. The findings have shown a relationship between anxiety, mood, and sleep disorders and PMP in women with breast cancer [33] (Table 1). The role of psychological risk factors is also supported by other findings reporting an association between pain and high levels of anxiety and depression [34,35]. A longitudinal study involving 410 women undergoing mastectomy showed that anxiety and depression before surgery were followed by moderate to severe persistent PMP [36] (Table 1). It is known that psychological stress can cause greater sensitivity to pain [37]. As a counterweight, it has recently been proposed that this major sensitivity increases attention to pain and motivates behaviors promoting and favoring healing [38]. In this context, an increase in the cortisol level has been shown to be accompanied by a decrease in prolactin occurring about 40 min after surgery, associated simultaneously with a significant postoperative decrease in lymphocytary glycoproteins CD4, CD8, and neuronal glycoprotein CD56 [39] (Table 1). Cortisol is a steroid hormone related to stress, produced by the adrenal gland cortex [40]. Contradictory results have been produced about the role of the cortisol level as a risk factor predicting the development of post-mastectomy pain. A study examined the relationship between demographic, clinical, psychological, and neuro-endocrinological factors and the development of post-surgery pain occurring in 64 women after partial mastectomy. In this sample of women, levels of anxiety and depression were evaluated before surgery through the Hospital Anxiety and Depression Scale (HADS). Cortisol levels in 24 h samples of urine were quantified two days before mastectomy. In the same sample of women, PMP was evaluated through a visual analogic scale (VAS) in the two days after surgery and using a short-form McGill Pain Questionnaire for chronic pain administered after six months. The results of the study showed an increase in risk for moderate to severe acute PMP associated with low levels of cortisol before surgery (odds ratio (95% confidence interval): 0.96 (0.92–0.98)). Moreover, the data indicated that women with a higher preoperative degree of anxiety and moderate to severe acute pain were more likely to develop chronic postoperative pain (OR (95% CI): 1.63 (1.23–2.40) and 5.07 (1.30–24.6)). Based on these results, the authors concluded that low perioperative cortisol secretion might be related to acute postoperative pain, but insufficient evidence was produced to assert that it contributes to the development of PMP [41] (Table 1). Different types of sensitivity disturbances such as allodynia, hyperalgesia, and paresthesia, either primary or as sequelae to other surgical procedures, have been proposed to be associated with the development of PMP [42]. A repeated cross-sectional survey was carried out in Denmark on 2411 patients who were subjected to mastectomy for primary breast cancer by examining self-evaluation pain reports including location, intensity, frequency of pain, and prevalence of sensory disturbances. All participants who experienced a mastectomy in the years 2005 and 2006 were subsequently examined in 2008 and again, using the same questionnaire, in 2012. In this sample, the prevalence of chronic pain after mastectomy ranged between 22% and 53%, and half of them reported sensory disturbances. Potential risk factors for chronic pain revealed by this research were axillary lymph node dissection rather than sentinel lymph node biopsy (odds ratio 2.04, 95% confidence interval 1.60 to 2.61; *p* < 0.001) and age ≤ 49 years (1.78, 1.25 to 2.54; *p* < 0.001). The results confirmed that an axillary procedure is a risk factor that can predict subsequent persistent pain and sensory disturbances [7] (Table 1). Pain has been related to treatment with chemotherapy and radiotherapy. This view is not sufficiently supported, but data published from a pilot study indicate that pain after mastectomy could be due to nerve injury during breast cancer surgery and radiotherapy [43]. Concerning age as a risk factor, it has been suggested that younger women with a history of chronic pain may be at a higher risk of developing PMP. A survey investigating the prevalence and risk factors of PMP, including 420 women, found that 22.4% of the sample was affected by chronic pain. Patients with chronic pain were younger than those not suffering from chronic pain. Moreover, multivariate analysis showed that age and history of chronic pain were independent risk factors [44] (Table 1). These aspects also emerged from a more recent investigation. The risk factors of 2033 women who underwent mastectomy were investigated in a retrospective study by asking them about the occurrence of chronic PMP. Based on the answers of 97.5% of responders, it emerged that 28.2% had developed chronic pain; using univariate analysis, age 35 years, tumor staging, previous chronic pain, total mastectomy, and axillary lymph node dissection were statistically associated with PMP [45] (Table 1). To explain the mechanisms through which younger age represents a risk factor for PMP, enhanced likelihood of a higher histopathological tumor grade and the need for adjuvant chemotherapy when the tumor occurs in younger women have been highlighted. As another possible factor, differences in estrogen receptor status in younger versus older patients have been evoked, together with the reduction in sensitivity of pain receptors in older women [15,46]. Factors potentially associated with chronic pain occurring in patients who underwent mastectomy for breast cancer were investigated through a systematic review and meta-analysis performed on 30 studies, including 19,813 patients. Analysis of the data confirmed an increase in the odds of chronic pain with younger age (OR for every 10-year decrement 1.36, 95% confidence interval (CI) 1.24–1.48) and radiotherapy (OR 1.35, 95% CI 1.16–1.57). Furthermore, the risk increased when axillary lymph node dissection (OR 2.41, 95% CI 1.73–3.35) was performed, leading to women being affected by more severe acute postoperative pain (OR for every 1 cm on a 10 cm VAS 1.16, 95% CI 1.03–1.30) and, albeit with moderate evidence, greater preoperative pain. The results did not show any relationship between persistent pain and body mass index, type of surgery, chemotherapy, or endocrine therapy [47] (Table 1).

Recent evidence supports the use of regional anesthesia for postoperative pain [48]. The potential benefits of regional techniques in breast surgery to avoid post-mastectomy pain were also investigated in an observational study recruiting 123 patients who underwent mastectomy. In this study, regional anesthesia produced lower acute post-mastectomy pain and a reduction in opioid administration in women with higher baseline pain catastrophising, measured via the Pain Catastrophizing Scale (a scale validated to measure catastrophic thinking associated with pain) [49].

It has been shown that paravertebral blocks, used for other regional methodologies in anesthesia, produce a diminution of the development of postsurgical pain [50,51]. However, a recent meta-analysis of 16 studies involving 1026 patients concluded that there was moderate- to high-level evidence that pectoral nerves blocks reduce PMP compared with no regional technique and cause a reduction in the frequency of post-surgery nausea and vomiting [52]. About this, a prospective cohort study, designed to identify factors predicting PMP, recruited 537 women who would subsequently undergo primary breast cancer surgery. According to the study results, the authors concluded that, on the basis of risk factors related to the patient and type of surgery performed, the occurrence of PMP could be predicted [53] (Table 1). According to their view, younger age with locoregional pain before surgery, together with axillary lymph node dissection and acute PMP with high intensity and signs of neuropathic pain in the acute post-surgery phase, should be considered to indicate high risk. Thus, identifying patients presenting these characteristics may ease adequate actions, consequently reducing PMP or its intensity.

#### 3.2.2. Genetic Predisposing Factors

Arising evidence indicates that the inherited factors influencing the occurrence of post-surgery chronic pain occur in about 45% in the general population and that genetics only incompletely explains individual predisposition to the development of chronic pain after surgical treatment [54]. However, it has been shown that gene polymorphism affects the efficacy and dosage of opioids [55], and it is thought to be a pre-surgery risk factor for the development of PMP. The research focused principally on the Mu1 receptor gene encoding for Mu1 opioid receptor and its involvement in pain modulation following surgery. The findings suggest that the individual variation observed in the occurrence of PMP is partially influenced by genetic variability in the gene locus for Mu1 opioid receptor (OPRM1). OPRM1 has a common variant called A118G (rs1799971), so named because this single-nucleotide polymorphism in exon 1 of the gene is responsible for the transition of an adenine (A) nucleotide to guanine (G) at base 118 [56]. It has been suggested that patients presenting the OPRM1 genotype may be at higher risk for chronic post-surgery pain because of their variability in response to opioids; OPRM1 could be monitored for the personalization of opioid treatment, thus avoiding chronicization of pain [11] (Table 1).

#### 3.2.3. Markers

To develop a clinical tool that can be used to identify risk factors for PMP, a prediction model was recently tested on women with operable unilateral breast cancer. Data were collected from Finland (*n* = 860), Denmark (*n* = 453), and Scotland (*n* = 231). The results of the test showed that pain before surgery, high body mass index, axillary lymph node dissection, and more severe acute postoperative pain can be considered predictors for chronic pain after mastectomy [13] (Table 1). Polymorphism in the gene CACNG2 has also been associated with the development of chronic PMP. It has been reported that the presence of specific alleles of single-nucleotide polymorphism and a 3-SNP haplotype (A-C-C) in the human gene CACNG2 (encoding for the voltage-dependent calcium channel gamma subunit 2 or stargazin) enhances the risk of pain chronicization after mastectomy due to breast cancer [10]. A more recent meta-analysis conducted on two cohorts of women confirmed a relationship between polymorphism in the gene CACNG2 and chronic pain after breast surgery. The authors of the meta-analysis concluded that the A-C-C haplotype at three single-nucleotide polymorphisms (rs4820242, rs2284015, and rs2284017) in the CACNG2 gene is related to enhanced risk of PMP [57] (Table 1). It is well known that catecholamines influence pain perception [58], and polymorphisms of genes for these substances have been associated with PMP. Inhibition of noradrenaline reuptake increases analgesia, acting via α2-adrenergic receptors located in the dorsal horn of the spinal cord. These receptors are coupled to the inhibitory G protein, which, in turn, blocks the presynaptic voltage-controlled calcium channels in the dorsal horn of the spinal cord, inhibiting the release of excitatory neurotransmitters from primary afferent fibers [59]. In particular, the polymorphisms that have been correlated with severe pain are the following: catechol-O-methyltransferase (COMT) high pain sensitivity (HPS) haplotype, the solute carrier family 6 (SLC6) member 2-noradrenaline transporter (SLC6A2) HapD01, and SLC6 member 3-noradrenaline transporter (SLC6A3) rs464049 [60] (Table 1). COMT is an enzyme inactivating catecholamines such as dopamine, noradrenaline, and adrenaline [61]. The Val158Met polymorphism, a common genetic variant in Caucasian populations, influences the activity of the COMT enzyme, and it has been suggested that interactions between OPRM1 and COMT might contribute to the development of post-surgery pain and sensitivity to opioids [62]. The SLC6 family of the human genome includes transporters for neurotransmitters, and they have a crucial role in neurotransmission [63]. Glycine transporters are Na^+^-/Cl^−^-dependent transporters of the SLC6 family, and it has been suggested that their inhibition could be a mechanism to increase spinal extracellular glycine concentrations, enhancing neurotransmission mediated by glycine receptors in the dorsal horn [64]. As the activation of Mu opioid receptors is involved in the modulation of pain, the study of these structures is relevant in the discovery of putative biomarkers to be used in the management of pain. In particular, Mu1 receptor is considered one main target in the context of analgesia with opioids. It is important to note that polymorphism of the gene encoding for Mu opioid receptor can influence the response to analgesic drugs in patients with cancer [65]. Among the variants, as reported above, the most frequent is SNP A118G. This genotype can be used as a biomarker for analgesia management after surgery in cancer patients, particularly in patients with cancer homozygous for the G allele of SNP A118G (rs1799971), generally requiring higher doses of morphine for the treatment of chronic pain [66]. Mediators of inflammation play a role in the development of chronic pain after surgery, participating in the beginning and subsequently maintaining chronic pain after nerve lesion through changes in activity, connectivity, and number of neuronal cells. The findings indicate the involvement of polymorphisms of the IL-6, C-X-C motif chemokine ligand 8 (CXCL8), and TNF genes and modification of methylation in the TNF gene promoter favoring the occurrence of chronic breast pain after breast cancer surgery [67] (Table 1).

## 4. Discussion

Chronic post-surgery pain is defined as ongoing pain directly imputable to a surgical procedure, persisting for at least three months after surgery [69]. Chronic neuropathic pain is chronic pain caused by a lesion or disease of the somatosensory system, and neuropathic modifications are generally associated with the development of chronic pain [7]. Neuropathic pain is frequent in the early period after breast cancer diagnosis [70]. The neuropathic origin of this pain can be considered one of the principal causes of disability and a fundamental stress factor in women affected by breast cancer patients, and it represents the most important contributor to the development of chronic breast pain [68] (Table 1). Nevertheless, chronic post-surgery pain should not be considered to be strictly related to only neuropathic pain. It is known that chronic pain occurrence is very frequent when surgical procedures involve major nervous injury or when nerve transection happens. For this reason, appropriate care can make the difference in avoiding chronicization of symptoms [19]. Only an accurate diagnosis can theoretically lead to the application of the proper pain management strategies, and this is particularly true for neuropathic pain, which is generated by nervous system lesions or dysfunction and can be maintained by several different mechanisms. Moreover, neuropathic pain is more likely to be caused by either surgical treatment or comorbidity, and it is more difficult to treat than nociceptive pain [71]. In this regard, it has been suggested that chronic pain occurring after breast cancer surgery may be produced by nerve damage in the axilla and/or chest wall owing to excision surgery [72] and possibly aggravated by successive chemotherapy and radiotherapy [40]. Generally, the principal predictive factor for post-surgery pain is the type of intervention, followed by preoperative pain, anxiety, and age [73]. Our overview of risk factors indicates that psychological factors play a role in the development of PMP. In particular, it has been observed that anxiety, mood, and sleep disorders are frequent pre-existing factors. A pre-surgery state of anxiety was also found to be accompanied by acute pain. Both these factors could be considered as predictors for PMP. Hormonal changes related to stress have been detected after breast surgery, consisting of increased cortisol and decreased prolactin levels. These modifications were found to be associated with a significant decrease in CD4, CD8, and CD56. In addition, sensory disorders have also been suggested to be related to PMP, together with axillary lymph node dissection and age of ≤49 years in women. In this context, younger age among women has been reported to be a higher risk factor for chronic pain after breast surgery. Gene polymorphism is a preoperative risk factor influencing the management of pain after breast cancer surgery. Genetic predisposition is characterized by the changeability of variant A1118G for Mu1 opioid receptor, causing an unpredictable response to opioids. Knowledge of this variant might be taken into account to customize treatment with opioids, thus reducing side effects.

## 5. Conclusions

Research on risk factors and biomarkers for PMP has produced crucial results that physicians conducting breast cancer surgeries should know about. First of all, investigation of individual polymorphisms of the gene CACNG2 can be useful in predicting the development of PMP. Other polymorphisms that could be investigated are related to catecholamines (COMT HPS haplotype, SLC6A2 HaplotypeD01, and SLC6A3 rs464049). Based on the involvement of Mu opioid receptors in pain perception, a study of variant SNPA118G could be performed to identify women homozygous for the G allele of SNPA118G (rs1799971), in which higher doses of opioids are needed to obtain analgesia. Finally, the study of polymorphisms of inflammatory mediators such as the IL6, CXCL8, and TNF genes deserves attention because it can be helpful in the choice of the proper pain therapy for the postoperative period following breast cancer surgery. PMP syndrome is a negative consequence occurring after breast cancer surgery. The identification of women most at risk is fundamental to setting up the best treatment to prevent this outcome. Clinical practice can be planned through the interpretation of genotyping data, choice of drugs, and tailoring of doses for each patient with the aim to provide safer and more effective individual analgesic treatment.

## Figures and Tables

**Table 1:** Risk factors, biomarkers, and associated factors involved in the development of chronic pain occurring in women after breast cancer surgery.

Surgery	Risk Factors and Biomarkers	Associated Factors	Postoperative Pain Perception	Source
Lumpectomy, mastectomy, or mastectomy with reconstruction	Pre-existing surgical area pain, less schooling, frequent somatization, sleep disorders, and ALND	Not reported	↑	Schreiber et al. 2021 [9]
Total or partial mastectomy	Anxiety, mood, and sleep disorders	Not reported	↑	Belfer et al. 2013 [33]
Radical mastectomy	↑ cortisol level ↓ prolactin ↓ CD4, CD8, CD56	Not reported	↑	Bakr et al. 2016 [39]
Partial mastectomy	↓ cortisol level	Higher level of anxiety and moderate to severe acute pain	↑	Nishimura et al. 2017 [41]
Unilateral mastectomy	ALND	Not reported	↑	Mejdahl et al. 2013 [8]
Mastectomy	History of chronic pain and younger age at surgery	Tumor staging, number of lymphadenectomy	↑	Cui et al. 2018 [44]
Unilateral mastectomy	Age ≥ 35 years, history of chronic pain, total mastectomy, and ALND	Tumor staging, previous chronic pain experience, total mastectomy	↑	Gong et al. 2020 [45]
Breast cancer surgery	Younger age, radiotherapy.	ALND, preoperative pain	↑	Wang et al. 2016 [47]
Breast cancer surgery	Age < 65 years, locoregional pain before surgery	Axillary lymph node dissection, breast conserving surgery, signs of neuropathic pain	↑	Andersen et al. 2015 [53]
Unilateral surgery	Moderate arm/shoulder pain in the first six months following breast cancer surgery	Younger age, higher BMI, higher number of lymph nodes dissection, ALND, anxiety, and depression	↑	Miaskowski et al. 2014 [36]
Breast cancer surgery	OPRM1 polymorphism	Not reported	Not reported	De Gregori et al. 2015 [11]
Breast cancer surgery	Neuropathic pain signs	Not reported	↑	Haroutiunian et al. 2013 [68]
Unilateral surgery	High body mass index, axillary lymph node dissection, postoperative pain intensity at the seventh postoperative day	Not reported	↑	Meretoja et al. 2017 [13]
Unilateral mastectomy or lumpectomy, accompanied by ALND	CACNG2 polymorphism	Chronic pain	PMP was present in 39% (215/549) of the study participants	Nissenbaum et al. 2010 [10]
Unilateral (or partial) surgery; unilateral; bilateral complete mastectomy or partial mastectomy	CACNG2 polymorphism	Not reported	PMP was present in 45% (216/482) of the study participants	Bortsov et al. 2019 [57]
Breast cancer surgery	COMT HPS haplotype, SLC6A2 HapD01, SLC6A3 rs464049 polymorphisms	Chronic pain	Not reported	Knisely et al. 2018 [60]
Unilateral breast surgery	IL6, CXCL8, TNF genes polymorphisms, and modification of methylation in the TNF gene promoter	Younger age (premenopausal), higher number of breast biopsies, presence of pain, strange sensations, and hardness in the breast prior to surgery, reconstructive surgery, re-excision or mastectomy in the 6 months following surgery	↑	Stephens et al. 2017 [67]

↑ increasing; ↓ decreasing; OPRM1 = Mu1 opioid receptor; CACNG2 = calcium voltage-gated channel auxiliary subunit gamma 2; COMT HPS = catechol-O-methyltransferase high pain sensitivity; SLC6A2 = solute carrier family 6 member 2-noradrenaline transporter; SLC6A3 = solute carrier family 6 member 3-noradrenaline transporter; CXCL8 = C-X-C motif chemokine ligand 8; TNF = tumor necrosis factor; ALND = axillary lymph node dissection; BMI = body mass index; PMP = post mastectomy pain.

## Data Availability

All data supporting the reported results can be found in the manuscript.

## References

[1] Siegel R.L., Miller K.D., Fuchs H.E., Jemal A. (2021). Cancer Statistics. CA Cancer J. Clin..

[2] Hanley M.A., Jensen M.P., Smith D.G., Ehde D.M., Edwards W.T., Robinson L.R. (2007). Preamputation pain and acute pain predict chronic pain after lower extremity amputation. J. Pain.

[3] Smith H.S., Wu S.X. (2012). Persistent pain after breast cancer treatment. Ann. Palliat. Med..

[4] Brummett C.M. (2011). Chronic pain following breast surgery. Tech Reg. Anesth. Pain Manag..

[5] Wang L., Cohen J.C., Devasenapathy N., Hong B.Y., Kheyson S., Lu D., Oparin Y., Kennedy S.A., Romerosa B., Arora N. (2020). Prevalence and intensity of persistent post-surgical pain following breast cancer surgery: A systematic review and meta-analysis of observational studies. Br. J. Anaesth..

[6] Andersen K.G., Kehlet H. (2011). Persistent pain after breast cancer treatment: A critical review of risk factors and strategies for prevention. J. Pain.

[7] Scholz J., Finnerup N.B., Attal N., Aziz Q., Baron R., Bennett M.I., Benoliel R., Cohen M., Cruccu G., Davis K.D. (2019). Classification Committee of the Neuropathic Pain Special Interest Group (NeuPSIG). The IASP classification of chronic pain for ICD-11: Chronic neuropathic pain. Pain.

[8] Mejdahl M.K., Andersen K.G., Gärtner R., Kroman N., Kehlet H. (2013). Persistent pain and sensory disturbances after treatment for breast cancer: Six year nationwide follow-up study. BMJ.

[9] Schreiber K.L., Zinboonyahgoon N., Flowers K.M., Hruschak V., Fields K.G., Patton M.E., Schwartz E., Azizoddin D., Soens M., King T. (2021). Prediction of Persistent Pain Severity and Impact 12 Months after Breast Surgery Using Comprehensive Preoperative Assessment of Biopsychosocial Pain Modulators. Ann. Surg. Oncol..

[10] Nissenbaum J., Devor M., Seltzer Z., Gebauer M., Michaelis M., Tal M., Dorfman R., Abitbul-Yarkoni M., Lu Y., Elahipanah T. (2010). Susceptibility to chronic pain following nerve injury is genetically affected by CACNG2. Genome Res..

[11] De Gregori M., Diatchenko L., Belfer I., Allegri M. (2015). OPRM1 receptor as new biomarker to help the prediction of post mastectomy pain and recurrence in breast cancer. Minerva Anestesiol..

[12] Mustonen L., Vollert J., Rice A.S.C., Kalso E., Harno H. (2020). Sensory profiles in women with neuropathic pain after breast cancer surgery. Breast Cancer Res. Treat..

[13] Meretoja T.J., Andersen K.G., Bruce J., Haasio L., Sipilä R., Scott N.W., Ripatti S., Kehlet H., Kalso E. (2017). Clinical Prediction Model and Tool for Assessing Risk of Persistent Pain After Breast Cancer Surgery. J. Clin. Oncol..

[14] Macrae W.A. (2008). Chronic post-surgical pain: 10 years on. Br. J. Anaesth..

[15] Tait R.C., Zoberi K., Ferguson M., Levenhagen K., Luebbert R.A., Rowland K., Salsich G.B., Herndon C. (2018). Persistent Post-Mastectomy Pain: Risk Factors and Current Approaches to Treatment. J. Pain.

[16] Blyth F.M., March L.M., Brnabic A.J., Cousins M.J. (2004). Chronic pain and frequent use of health care. Pain.

[17] Nazarnia S., Subramaniam K. (2021). Nonopioid Analgesics in Postoperative Pain Management after Cardiac Surgery. Semin. Cardiothorac. Vasc. Anesth..

[18] Carroll I., Hah J., Mackey S., Ottestad E., Kong J.T., Lahidji S., Tawfik V., Younger J., Curtin C. (2013). Perioperative interventions to reduce chronic postsurgical pain. J. Reconstr. Microsurg..

[19] Humble S.R., Dalton A.J., Li L. (2015). A systematic review of therapeutic interventions to reduce acute and chronic post-surgical pain after amputation, thoracotomy or mastectomy. Eur. J. Pain.

[20] Chappell A.G., Bai J., Yuksel S., Ellis M.F. (2020). Post-Mastectomy Pain Syndrome: Defining Perioperative Etiologies to Guide New Methods of Prevention for Plastic Surgeons. World J. Plast Surg..

[21] Miller A.H., Ancoli-Israel S., Bower J.E., Capuron L., Irwin M.R. (2008). Neuroendocrine-immune mechanisms of behavioral comorbidities in patients with cancer. J. Clin. Oncol..

[22] Dantzer R., O’Connor J.C.F., Freund G.G., Johnson R.W., Kelley K.W. (2008). From inflammation to sickness and depression: When the immune system subjugates the brain. Nat. Rev. Neurosci..

[23] Arnow B.A., Hunkeler E.M., Blasey C.M., Lee J., Constantino M.J., Fireman B., Kraemer H.C., Dea R., Robinson R., Hayward C. (2006). Comorbid depression, chronic pain, and disability in primary care. Psychosom. Med..

[24] Leonard B.E. (2015). Pain, Depression and Inflammation: Are Interconnected Causative Factors Involved?. Mod. Trends Pharm..

[25] Thornton L.M., Andersen B.L., Blakely W.P. (2010). The pain, depression, and fatigue symptom cluster in advanced breast cancer: Covariation with the hypothalamic-pituitary-adrenal axis and the sympathetic nervous system. Health Psychol..

[26] Starkweather A.R., Lyon D.E., Schubert C.M. (2013). Pain and inflammation in women with early-stage breast cancer prior to induction of chemotherapy. Biol. Res. Nurs..

[27] Poquet N., Lin C. (2016). The Brief Pain Inventory (BPI). J. Physiother..

[28] Fazzari J., Sidhu J., Motkur S., Inman M., Buckley N., Clemons M., Vandermeer L., Singh G. (2020). Applying Serum Cytokine Levels to Predict Pain Severity in Cancer Patients. J. Pain Res..

[29] Lee E., Nelson O.L., Puyana C., Takita C., Wright J.L., Zhao W., Reis I.M., Lin R.Y., Hlaing W.M., Bakalar J.L. (2019). Association between C-reactive protein and radiotherapy-related pain in a tri-racial/ethnic population of breast cancer patients: A prospective cohort study. Breast Cancer Res..

[30] Hjermstad M.J., Fayers P.M., Haugen D.F., Caraceni A., Hanks G.W., Loge J.H., Fainsinger R., Aass N., Kaasa S. (2011). European Palliative Care Research Collaborative (EPCRC). Studies comparing Numerical Rating Scales, Verbal Rating Scales, and Visual Analogue Scales for assessment of pain intensity in adults: A systematic literature review. J. Pain Symptom Manag..

[31] Calapai F., Mondello E., Mannucci C., Sorbara E.E., Gangemi S., Quattrone D., Calapai G., Cardia L. (2021). Pain Biomarkers in Cancer: An Overview. Curr. Pharm. Des..

[32] Schreiber K.L., Martel M.O., Shnol H., Shaffer J.R., Greco C., Viray N., Taylor L.N., McLaughlin M., Brufsky A., Ahrendt G. (2013). Persistent pain in postmastectomy patients: Comparison of psychophysical, medical, surgical, and psychosocial characteristics between patients with and without pain. Pain.

[33] Belfer I., Schreiber K.L., Shaffer J.R., Shnol H., Blaney K., Morando A., Englert D., Greco C., Brufsky A., Ahrendt G. (2013). Persistent postmastectomy pain in breast cancer survivors: Analysis of clinical, demographic, and psychosocial factors. J. Pain.

[34] Hinrichs-Rocker A., Schulz K., Järvinen I., Lefering R., Simanski C., Neugebauer E.A. (2009). Psychosocial predictors and correlates for chronic post-surgical pain (CPSP)—A systematic review. Eur. J. Pain..

[35] Masselin-Dubois A., Attal N., Fletcher D., Jayr C., Albi A., Fermanian J., Bouhassira D., Baudic S. (2013). Are psychological predictors of chronic postsurgical pain dependent on the surgical model? A comparison of total knee arthroplasty and breast surgery for cancer. J. Pain.

[36] Miaskowski C., Paul S.M., Cooper B., West C., Levine J.D., Elboim C., Hamolsky D., Abrams G., Luce J., Dhruva A. (2014). Identification of patient subgroups and risk factors for persistent arm/shoulder pain following breast cancer surgery. Eur. J. Oncol. Nurs..

[37] Crettaz B., Marziniak M., Willeke P., Young P., Hellhammer D., Stumpf A., Burgmer M. (2013). Stress-induced allodynia--evidence of increased pain sensitivity in healthy humans and patients with chronic pain after experimentally induced psychosocial stress. PLoS ONE.

[38] Timmers I., Kaas A.L., Quaedflieg C.W.E.M., Biggs E.E., Smeets T., de Jong J.R. (2018). Fear of pain and cortisol reactivity predict the strength of stress-induced hypoalgesia. Eur. J. Pain.

[39] Bakr M.A., Amr S.A., Mohamed S.A., Hamed H.B., Abd El-Rahman A.M., Mostafa M.A., El Sherif F.A. (2016). Comparison Between the Effects of Intravenous Morphine, Tramadol, and Ketorolac on Stress and Immune Responses in Patients Undergoing Modified Radical Mastectomy. Clin. J. Pain.

[40] Úbeda-D’Ocasar E., Jiménez Díaz-Benito V., Gallego-Sendarrubias G.M., Valera-Calero J.A., Vicario-Merino Á., Hervás-Pérez J.P. (2020). Pain and Cortisol in Patients with Fibromyalgia: Systematic Review and Meta-Analysis. Diagnostics.

[41] Nishimura D., Kosugi S., Onishi Y., Ihara N., Wakaizumi K., Nagata H., Yamada T., Suzuki T., Hashiguchi S., Morisaki H. (2017). Psychological and endocrine factors and pain after mastectomy. Eur. J. Pain.

[42] Gärtner R., Jensen M.B., Nielsen J., Ewertz M., Kroman N., Kehlet H. (2009). Prevalence of and factors associated with persistent pain following breast cancer surgery. JAMA.

[43] Hojan K., Wojtysiak M., Huber J., Molińska-Glura M., Wiertel-Krawczuk A., Milecki P. (2016). Clinical and neurophysiological evaluation of persistent sensory disturbances in breast cancer women after mastectomy with or without radiotherapy. Eur. J. Oncol. Nurs..

[44] Cui L., Fan P., Qiu C., Hong Y. (2018). Single institution analysis of incidence and risk factors for post-mastectomy pain syndrome. Sci. Rep..

[45] Gong Y., Tan Q., Qin Q., Wei C. (2020). Prevalence of postmastectomy pain syndrome and associated risk factors: A large single-institution cohort study. Medicine.

[46] Meijuan Y., Zhiyou P., Yuwen T., Ying F., Xinzhong C. (2013). A retrospective study of postmastectomy pain syndrome: Incidence, characteristics, risk factors, and influence on quality of life. Sci. World J..

[47] Wang L., Guyatt G.H., Kennedy S.A., Romerosa B., Kwon H.Y., Kaushal A., Chang Y., Craigie S., de Almeida C.P.B., Couban R.J. (2016). Predictors of persistent pain after breast cancer surgery: A systematic review and meta-analysis of observational studies. CMAJ.

[48] Curatolo M. (2016). Regional anesthesia in pain management. Curr. Opin. Anaesthesiol..

[49] Zinboonyahgoon N., Patton M.E., Chen Y.K., Edwards R.R., Schreiber K.L. (2021). Persistent Post-Mastectomy Pain: The Impact of Regional Anesthesia Among Patients with High vs. Low Baseline Catastrophizing. Pain Med..

[50] Tahiri Y., Tran D.Q., Bouteaud J., Xu L., Lalonde D., Luc M., Nikolis A. (2011). General anaesthesia versus thoracic paravertebral block for breast surgery: A meta-analysis. J. Plast Reconstr. Aesthet. Surg..

[51] Shimizu H., Kamiya Y., Nishimaki H., Denda S., Baba H. (2015). Thoracic paravertebral block reduced the incidence of chronic postoperative pain for more than 1 year after breast cancer surgery. JA Clin. Rep..

[52] Grape S., Jaunin E., El-Boghdadly K., Chan V., Albrecht E. (2020). Analgesic efficacy of PECS and serratus plane blocks after breast surgery: A systematic review, meta-analysis and trial sequential analysis. J. Clin. Anesth..

[53] Andersen K.G., Duriaud H.M., Jensen H.E., Kroman N., Kehlet H. (2015). Predictive factors for the development of persistent pain after breast cancer surgery. Pain.

[54] Chidambaran V., Gang Y., Pilipenko V., Ashton M., Ding L. (2020). Systematic Review and Meta-Analysis of Genetic Risk of Developing Chronic Postsurgical Pain. J. Pain.

[55] Lu L. (2015). The impact of genetic variation on sensitivity to opioid analgesics in patients with postoperative pain: A systematic review and meta-analysis. Pain Phys..

[56] Mura E., Govoni S., Racchi M., Carossa V., Ranzani G.N., Allegri M., van Schaik R.H. (2013). Consequences of the 118A>G polymorphism in the OPRM1 gene: Translation from bench to bedside?. J. Pain Res..

[57] Bortsov A.V., Devor M., Kaunisto M.A., Kalso E., Brufsky A., Kehlet H., Aasvang E., Bittner R., Diatchenko L., Belfer I. (2019). CACNG2 polymorphisms associate with chronic pain after mastectomy. Pain.

[58] Martikainen I.K., Hagelberg N., Jääskeläinen S.K., Hietala J., Pertovaara A. (2018). Dopaminergic and serotonergic mechanisms in the modulation of pain: In vivo studies in human brain. Eur. J. Pharmacol..

[59] Obata H. (2017). Analgesic Mechanisms of Antidepressants for Neuropathic Pain. Int. J. Mol. Sci..

[60] Knisely M.R., Conley Y.P., Kober K.M., Smoot B., Paul S.M., Levine J.D., Miaskowski C. (2018). Associations Between Catecholaminergic and Serotonergic Genes and Persistent Breast Pain Phenotypes After Breast Cancer Surgery. J. Pain.

[61] Andersen S., Skorpen F. (2009). Variation in the COMT gene: Implications for pain perception and pain treatment. Pharmacogenomics.

[62] Palada V., Kaunisto M.A., Kalso E. (2018). Genetics and genomics in postoperative pain and analgesia. Curr. Opin. Anaesthesiol..

[63] Bröer S., Gether U. (2012). The solute carrier 6 family of transporters. Br. J. Pharmacol..

[64] Cioffi C.L. (2021). Inhibition of Glycine Re-Uptake: A Potential Approach for Treating Pain by Augmenting Glycine-Mediated Spinal Neurotransmission and Blunting Central Nociceptive Signaling. Biomolecules.

[65] Wei S.Y., Chen L.F., Lin M.W., Li W.C., Low I., Yang C.J., Chao H.T., Hsieh J.C. (2017). The OPRM1 A118G polymorphism modulates the descending pain modulatory system for individual pain experience in young women with primary dysmenorrhea. Sci. Rep..

[66] Yu Z., Wen L., Shen X., Zhang H. (2019). Effects of the OPRM1 A118G Polymorphism (rs1799971) on Opioid Analgesia in Cancer Pain: A Systematic Review and Meta-Analysis. Clin. J. Pain.

[67] Stephens K.E., Levine J.D., Aouizerat B.E., Paul S.M., Abrams G., Conley Y.P., Miaskowski C. (2017). Associations between genetic and epigenetic variations in cytokine genes and mild persistent breast pain in women following breast cancer surgery. Cytokine.

[68] Haroutiunian S., Nikolajsen L., Finnerup N.B., Jensen T.S. (2013). The neuropathic component in persistent postsurgical pain: A systematic literature review. Pain.

[69] Werner M.U., Kongsgaard U.E.I. (2014). Defining persistent post-surgical pain: Is an update required?. Br. J. Anaesth..

[70] Pereira S., Fontes F., Sonin T., Dias T., Fragoso M., Castro-Lopes J., Lunet N. (2017). Neuropathic Pain after Breast Cancer Treatment: Characterization and Risk Factors. J. Pain Symptom Manag..

[71] Mondello E., Quattrone D., Cardia L., Bova G., Mallamace R., Barbagallo A.A., Mondello C., Mannucci C., Di Pietro M., Arcoraci V. (2018). Cannabinoids and spinal cord stimulation for the treatment of failed back surgery syndrome refractory pain. J. Pain Res..

[72] Andersen K.G., Aasvang E.K., Kroman N., Kehlet H. (2014). Intercostobrachial nerve handling and pain after axillary lymph node dissection for breast cancer. Acta Anaesthesiol. Scand..

[73] Kehlet H., Jensen T.S., Woolf C.J. (2006). Persistent postsurgical pain: Risk factors and prevention. Lancet.

